# The Correlations of Clinical Outcomes and Vascular Morphology With Infarct Patterns in Middle Cerebral Arterial Occlusion

**DOI:** 10.1002/brb3.71226

**Published:** 2026-01-28

**Authors:** ZhiRong Cai, Yuan Chen, ShaoQing Pei, Yue He, YaNan Zhu, Rui Zhang, JingWei Lin, Yi Yang, Ying Zhu

**Affiliations:** ^1^ Department of Neurology Affiliated Hospital of Jiangsu University Zhenjiang Jiangsu China; ^2^ Department of Neurology Siyang Hospital Suqian Jiangsu China; ^3^ Department of Neurology Siyang Kangda Hospital Suqian Jiangsu China

**Keywords:** infarct pattern, large vessel occlusion, middle cerebral artery, vascular morphology

## Abstract

**Objective:**

To compare the clinical outcomes among various infarct patterns and to investigate the associations between the morphological parameters of contralateral middle cerebral artery (cMCA) M_1_ segment and infarct patterns in ischemic stroke attributed to large vessel occlusion (LVO) in M_1_ segment caused by intracranial atherosclerotic disease (ICAD).

**Methods:**

Patients with stroke attributed to M_1_‐ICAD‐LVO were enrolled. The infarct patterns were categorized into artery‐to‐artery embolism (AAE), large infarct, borderzone infarct (BZI), and perforating artery infarction (PAI). The morphological parameters of cMCA‐M_1_ segment included proximal and distal diameter, arc, and chord length. The tortuosity index of cMCA‐M_1_ segment was calculated by (arc length/chord length − 1) × 100%.

**Results:**

A total of 171 participants were enrolled. Compared to AAE, the risk of poor outcome increased in BZI (odds ratio [OR] = 5.51, 95% confidence interval [CI] = 1.71–17.78, *p* = 0.004) and large infarct (OR = 10.92, 95% CI = 2.01–59.27, *p* = 0.006) and was comparable in PAI. The tortuosity index (OR = 2.85, 95% CI = 1.13–7.18, *p* = 0.026) and arc length (OR = 2.47, 95% CI = 1.02–5.97, *p* = 0.045) significantly increased in BZI than other three patterns. Participants other than BZI were categorized into large infarct (*n* = 32) and non‐large‐infarct (*n* = 46) groups, and the proximal diameter (OR = 0.22, 95% CI = 0.07–0.72, *p* = 0.012), arc length (OR = 0.88, 95% CI = 0.78–0.98, *p* = 0.018), and chord length (OR = 0.87, 95% CI = 0.77–0.995, *p* = 0.042) were associated with large infarct.

**Conclusion:**

For patients with M_1_‐ICAD‐LVO, large infarct and BZI had poorer outcomes than PAI and AAE. The cMCA‐M_1_ segment with elevated tortuosity and arc length was associated with BZI, whereas a thin and short M_1_ segment was correlated with large infarct in patients with a less tortuous cMCA trunk.

## Graphical abstract

For stroke caused by large vessel occlusion in M_1_ segment of middle cerebral artery (MCA), a tortuous and long M_1_ segment of contralateral MCA (cMCA) correlates with the onset of borderzone infarct. In those with a less tortuous cMCA‐M_1_ segment, a thin and short M_1_ segment correlates with large infarct.

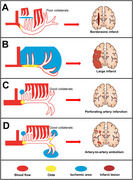

## 1 Introduction

Approximate 30% patients with ischemic stroke are attributed to large vessel occlusion (LVO), and this subtype of stroke has higher risks of poststroke mortality and disability than other subtypes (Waqas et al. 2020). Middle cerebral artery (MCA) trunk occlusion, namely, M_1_ segment occlusion, takes account for 1/3–1/2 patients with LVO stroke (Mokin et al. 2018; Waqas et al. 2020; Yang et al. 2024), and intracranial atherosclerotic disease (ICAD) is one of the common pathogenies of LVO (Lee et al. 2022), especially in Asian population (Jia et al. 2018).

In clinical practice, we have observed that ischemic stroke caused by M_1_‐ICAD‐LVO can be categorized into several patterns: artery‐to‐artery embolism (AAE), large infarct, border‐zone infarct (BZI), perforating artery infarction (PAI), and mixed pattern, consistent with some previous works (Lee et al. 2005; Feng et al. 2019; Tekle and Hassan 2021). What causes the heterogeneity of infarct patterns from ICAD‐LVO, rather than a uniform pattern with a large ischemic lesion in the affected territory? This maybe due to the different extents of collaterals between individuals (Faber et al. 2021). In case of occlusion in a major supply artery, the blood flow in the collaterals will be redistributed by the cerebral automatic regulation mechanism to sustain the supply for the ischemic core and peri‐infarct region (Binder et al. 2024). This compensated mechanism may contributes to provide partial flow to the territory of occlusive MCA thus decrease the ischemic volume (Elijovich et al. 2016) and ultimately represents with different infarct patterns.

The poststroke outcomes of these infarct patterns are with large disparities. The rate of poor clinical outcome is higher than 70% in patients with a large infarct even though with endovascular treatments (Yoshimura et al. 2022; Sarraj et al. 2023; Huo et al. 2023), whereas is approximately 20% in patients with BZI or PAI (Yong et al. 2006; Yang et al. 2023). However, the comparisons of poor outcomes among these patterns in the context of M_1_‐ICAD‐LVO are still unknown. Exploring the pathogenesis underlying the infarct patterns caused by M_1_‐ICAD‐LVO is needed because of the probable diversity of their clinical outcomes. According to previous reports, AAE is likely to be involved with the presence of vulnerable atherosclerotic plaque (Wu et al. 2018); a large infarct maybe introduced by a complete occlusion resulted from in situ thrombosis (Lee et al. 2005); BZI usually indicates insufficient blood flow in culprit vessel (Das et al. 2023); and PAI is associated with the blockage of the orifice of the perforating artery by a parental arterial atheroma (Caplan 2015; Yoon et al. 2013). In a word, the pathogenesis probably correlates with impaired hemodynamics in BZI and with the atherosclerotic plaque features of MCA in other three infarct patterns.

Vascular tortuosity can influence the progression of atherosclerotic plaque through its effects on hemodynamics (Brown et al. 2016), and a study has demonstrated the vascular tortuosity is associated with the presence of plaque in MCA (Kim et al. 2015). A recent study based on the high‐resolution vessel wall imaging has further found the vascular tortuosity involves with the plaque features (Li et al. 2024). In addition, the increased vascular tortuosity results in local turbulence in the vessels thereby impairs the territorial hemodynamics (Ha et al. 2023). Other than the vascular tortuosity, the luminal diameter can promote the plaque rupture and thrombosis (Richardson et al. 1989) through the impact on the plaque structural stress (Teng et al. 2014). In summary, vascular morphology seems to have an impact on the luminal plaque features and hemodynamics, thereby potentially associating with the infarct patterns caused by M_1_‐ICAD‐LVO.

This study compares the clinical outcomes of various infarct patterns attributed to M_1_‐ICAD‐LVO and investigates the relationship of these patterns with M_1_ segment morphology, aiming to preliminarily understand the clinical outcomes and potential pathogenesis of different infarct patterns and to provide some enlightenment for the clinical managements of M_1_‐ICAD patients with a high risk of acute occlusion and subsequent ischemic stroke.

## 2 Materials and Methods

### 2.1 Study Populations

This study is a double‐center cross‐sectional study that adheres to The Strengthening the Reporting of Observational Studies in Epidemiology (STROBE) statement and has been approved by The Scientific Research Ethics Committees of Affiliated Hospital of Jiangsu University and Siyang Hospital, respectively. All participants or their legal guardians provided informed consents. We screened all patients with acute or subacute ischemic stroke within 14 days of onset who were admitted to the stroke units of Affiliated Hospital of Jiangsu University and Siyang Hospital from August 01, 2020 to December 31, 2023, with the inclusion criteria: (1) ischemic stroke caused by M_1_‐ICAD‐LVO; (2) with an age of 18 years or older. Patients with potential cardioembolic source (e.g., atrial fibrillation, cardiomyopathy, and patent foramen ovale); with intravenous thrombolysis and/or endovascular treatment; with M_1_‐LVO due to non‐ICAD (e.g., dissection, vasculitis, and Moyamoya disease); with poor neuroimaging quality or lack of neuroimaging evidence; with occlusion in bilateral MCAs or anterior cerebral arteries (ACAs); with tandem stenosis in ipsilateral carotid artery; with a large chronic infarct in the territory of occlusive MCA; with functional dependence before the index stroke (i.e., modified Ranking Scale [mRS] 3–5 points) were excluded. PAI resulted from the blockage of the orifice of the perforating artery by ICAD in parental artery usually involves with the lowest section of the basal ganglia (Nah et al. 2010); thus, those without the infarct extending to the basal ganglia were also excluded.

On admission, all clinical data of subjects, including age, sex, a history of hypertension and diabetes mellitus, smoking, and a history of previous stroke (including both ischemic and hemorrhagic stroke), were collected by a face to face interview. The severity of neurological deficit of participants was evaluated by a neurologist via National Institute of Health Stroke Scale (NIHSS), and all participants were administrated according to the up‐to‐date guidelines (Warner et al. 2019; Kleindorfer et al. 2021). The daily activities of all participants were evaluated according to mRS (0–5 points, death was recorded as 6 points) at discharge. A poor clinical outcome referred to an mRS of 3–6 points at discharge.

### 2.2 The Infarct Patterns Evaluated via Neuroimaging

**FIGURE 1:**
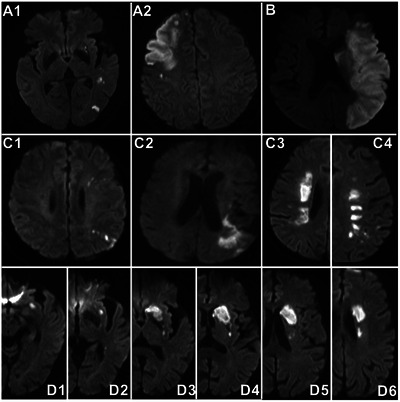
**Graphical representations of various infarct patterns caused by M_1_‐ICAD‐LVO**. (A1–A2) artery‐to‐artery embolism; (B) large infarct; (C1–C2) the cortical external border‐zone infarcts; (C3) the confluent internal border‐zone infarcts; (C4) the partial internal border‐zone infarcts; (D1–D6) perforating artery infarction. ICAD, intracranial atherosclerotic disease; LVO, large vessel occlusion.

The infarct patterns were categorized based on Chinese Ischemic Stroke Subclassification (Gao et al. 2011) as follows: (1) AAE: single or multiple small infarcts limited to the cortex, with or without small subcortical infarct(s). A large wedge‐ or oval‐shaped infarct limited to the cortex in the territory of MCA but not involved with the border‐zone was also categorized as AAE (Feng et al. 2019); (2) a large infarct: The volume of the ischemic lesion involved with both cortical and subcortical regions was 70 mL or greater in CT scan or diffused weighted image (Huo et al. 2023); (3) BZI: This pattern was divided into external cortical border‐zone and internal BZIs. The former referred to the linear‐, wedge‐, or oval‐shaped lesion(s) in the regions between the cortical territories of ACA and MCA or between MCA and posterior cerebral artery, the latter was defined as infarcts in the white matter along and (or) above the lateral ventricles between the deep and the superficial supplying territories of MCA or between the superficial territories of MCA and ACA, including confluent and partial subtypes. The confluent internal BZI represented as a large cigar shape, the partial internal BZI as a linear‐shaped lesion paralleling to the lateral ventricle with at least 3 lesions and each lesion with a diameter of 3 mm or larger (Lee et al. 2005; Feng et al. 2019); (4) PAI: single or multiple subcortical infarcts extending from the lowest portion of basal ganglia to radiating corona or semioval center (Feng et al. 2019; Nah et al. 2010). In case of multiple subcortical infarcts, the distribution of the lesion differed from the partial internal BZI (Figure 1).

Some scholars suggested the infarct patterns caused by MCA‐ICAD included a mixed pattern (Feng et al. 2019; Tekle and Hassan 2021). The mixed patterns of the subjects in this study represented in manner of BZI combined with AAE or PAI. The hypoperfusion could impair the clearance of emboli which would block the cortical or perforating arteries thus result in AAE or PAI (Sedlaczek et al. 2005). Therefore, we considered that the hypoperfusion played an important role in the mixed patterns, and these patterns were also categorized as BZI in this study, in accordance with a previous work (Quintero‐Consuegra et al. 2021).

Two independent neurologists evaluated the infarct patterns, respectively. The intra‐observer agreements (with *κ* values of 0.916 and 0.907, respectively, with both *p* values of <0.001) and the inter‐observer agreements (with a *κ* value of 0.860 and with a *p* value of <0.001) of the infarct pattern evaluation were excellent.

### 2.3 The MCA Morphology Measured via Magnetic Resonance or CT Angiography

The morphology of the culprit MCA could not be measured due to the complete occlusion. A previous study reported the morphological features of bilateral MCAs were with no significant differences in humans (Kim et al. 2015); we therefore measured the morphology of contralateral MCA (cMCA) to surrogate that of the culprit MCA.

The method of the measurement of the cMCA‐M_1_ segment morphology in this study was in line with a previous work (Kim et al. 2015). MCA‐M_1_ segment referred to the vessel from the bifurcation of ACA and MCA to the MCA bifurcation. We measured the morphological parameters of cMCA‐M_1_ segment, including the proximal diameter, the distal diameter, the arc length, and the chord length.

**FIGURE 2:**
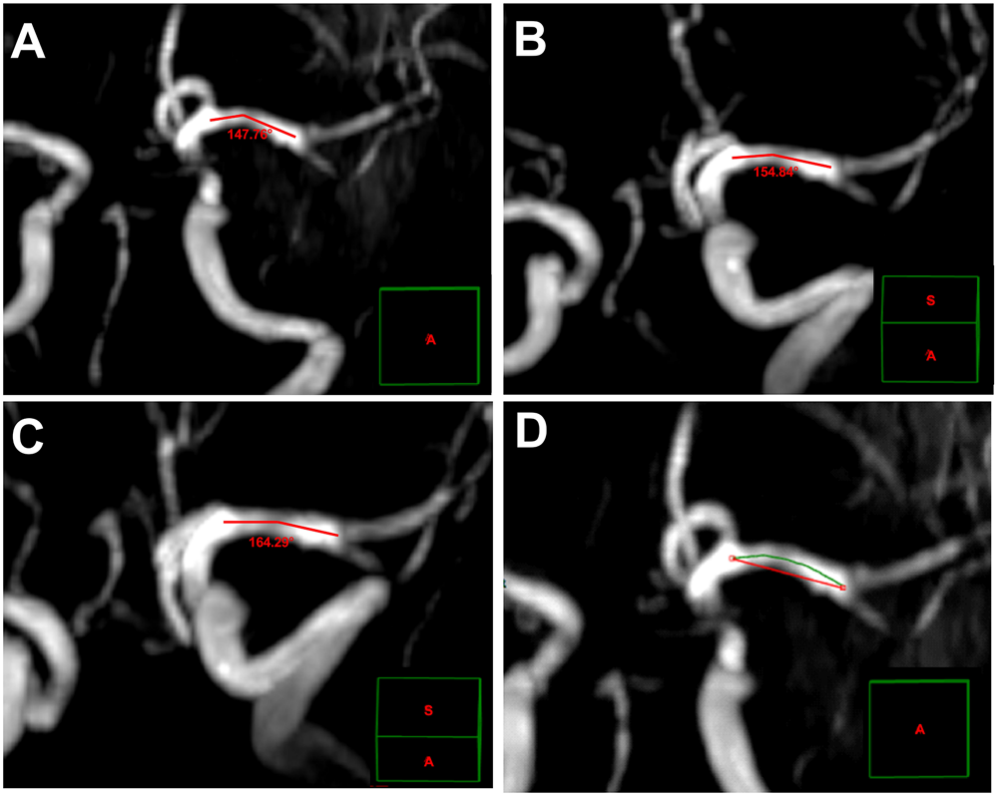
**The measurements of the morphological parameters in U‐shaped cMCA**. (A–C) The measurements of included angle (at the point where the two red lines met) of cMCA trunk; (D) the section with the smallest included angle (A) was used to measure the arc length (green line) and the chord length (red line). The green line box in the lower right corner of each image indicates the spatial position of this section in the forward three‐dimensional cerebrovascular imaging. cMCA, contralateral middle cerebral artery.

The shape of cMCA‐M_1_ segment majorly included U, S, and linear shapes (Kim et al. 2015). The morphological parameters of linear‐shaped cMCA were measured in any section with clearly exposed cMCA‐M_1_ segment chosen from the forward three‐dimensional cerebrovascular imaging in magnetic resonance or CT angiography. For U‐ and S‐shaped cMCA‐M_1_ segment, we rotated the three‐dimensional imaging along the horizontal axis and selected the section with the smallest included angle of cMCA‐M_1_ segment to measure the parameters. Two lines starting, respectively, from the ACA‐MCA bifurcation and MCA bifurcation and running along the central line of the vessel were drawn, and the angle at the point where these two lines met was the included angle of U‐shaped cMCA‐M_1_ segment (Figure 2).

**FIGURE 3:**
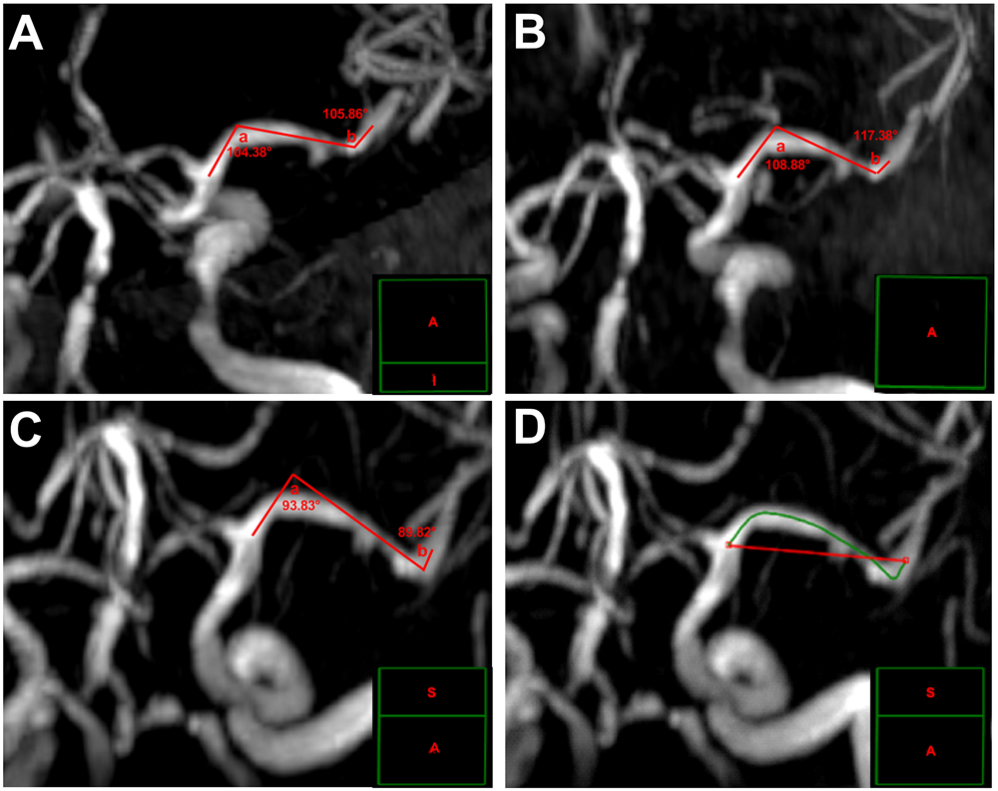
**The measurements of the morphological parameters in S‐shaped cMCA**. (A–C) The measurements of included angle (Angle a and b) of cMCA trunk; (D) the section with the smallest sum of included angles (C) was used to measure the arc length (green line) and the chord length (red line). The green line box in the lower right corner of each image indicates the spatial position of this section in the forward three‐dimensional cerebrovascular imaging. cMCA, contralateral middle cerebral artery.

For S‐shaped cMCA‐M_1_ segment, the included angles of two major turning points were measured. One angle was between the central line starting from ACA–MCA bifurcation and the central line of the mainstream of the middle part of the vessel (Angle a), whereas the latter and the central line starting from the MCA bifurcation formed the other angle (Angle b) (Figure 3). The section with the smallest sum of the two angles was selected. If the contralateral ACA‐A_1_ segment was lack, we estimated the approximate starting point of cMCA according to the site of ipsilateral ACA–MCA bifurcation.

The arc length was the length of the arc lying between the ACA–MCA bifurcation and the MCA bifurcation along the central line of the trunk, and the chord length referred to the minimal distance between the two bifurcations. The formula of the tortuosity index of cMCA‐M_1_ segment was (arc length/chord length − 1) × 100% (Kim et al. 2016). The proximal and distal diameters of cMCA‐M_1_ segment were measured within 2 mm from the two bifurcations, perpendicular to the direction of the trunk.

**FIGURE 4:**
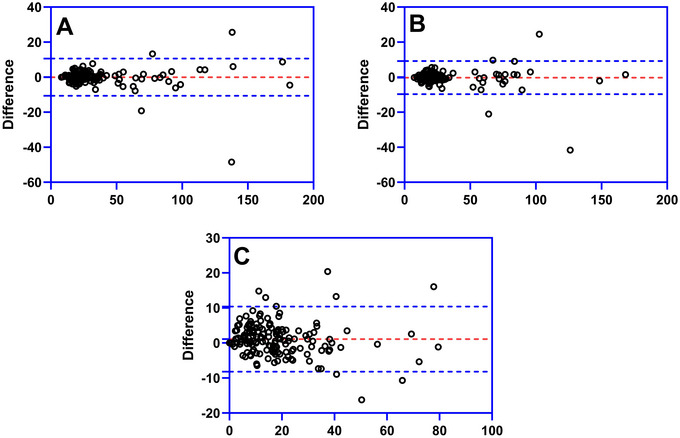
**Bland–Altman test in the assessments of inter‐observer agreements in the cMCA‐M_1_ morphological parameters measurements**. 97.7% of the proximal diameter (A), 97.1% of distal parameter (B), and 94.7% of tortuosity index (C) were within 95% limit of agreement in Bland–Altman test. cMCA, contralateral middle cerebral artery.

The work software Carestream RISGC (Carestream Health, USA, version 3.1.S05.0) was used to measure the morphological parameters of cMCA‐M_1_ segment. Two neurologists blinded to the clinical datum of all subjects, respectively, measured the morphological parameters of cMCA‐M_1_ segment, the mean of which was used in the statistical analyses. The inter‐observer agreements were acceptable in the measurements of morphological parameters (97.7% of the proximal diameter values, 97.1% of distal parameter, and 94.7% of tortuosity index were within 95% limit of agreement in Bland–Altman test) (Figure 4).

We also evaluated whether cMCA‐M_1_ segment had ICAD which was reported to be associated with the vascular morphology (Kim et al. 2015). ICAD referred to any degree of atherosclerotic stenosis in cMCA‐M_1_ segment. The stenotic degree was evaluated according to Warfarin‐Aspirin Symptomatic Intracranial Disease Study Trial method.

### 2.4 Statistical Methods

Software SPSS (version 25.0) was used to perform the statistical analyses. At first, we completed the missing data by using multiple imputation (five imputations) and chose the set of data with the highest Cronbach's alpha coefficient in reliability analysis. Normally distributed continuous variables were described by the mean ± standard deviation and non‐normally distributed continuous variables were described by the median (inter‐quartile range). The comparisons of categorical variables were performed by using chi‐squared test or Fisher's exact test. Kruskal–Wallis test or one‐way analysis of variance test was used to compare the continuous variables among multiple groups, and independent sample *t*‐test or Wilcoxon test was used to compare the continuous variables between two groups.

The participants were divided into four groups based on the infarct patterns subclassification and univariate analyses among these groups were performed. Age, sex, and confounders with a *p* value <0.2 were included into multivariable regression model. The binary logistic regression was used to analyze the association between the infarct pattern and poor clinical outcomes. According to the tertiles of the vascular morphological parameters with a significant difference in the univariable analyses, we trichotomized the participants respectively and analyzed the associations between the infarct patterns and the levels of these parameters in an ordinal logistic regression model. Due to the lowest rate of poor outcome, AAE group was used as the reference in multivariable analyses. Consequently, we divided the participants other than BZI patients into large infarct and non‐large‐infarct groups, and the binary logistic regression was used to analyze the association between the morphological parameters and the onset of large infarct. All tests were two sided and a *p* value of <0.05 was considered statistically significant.

## 3 Results

**FIGURE 5:**
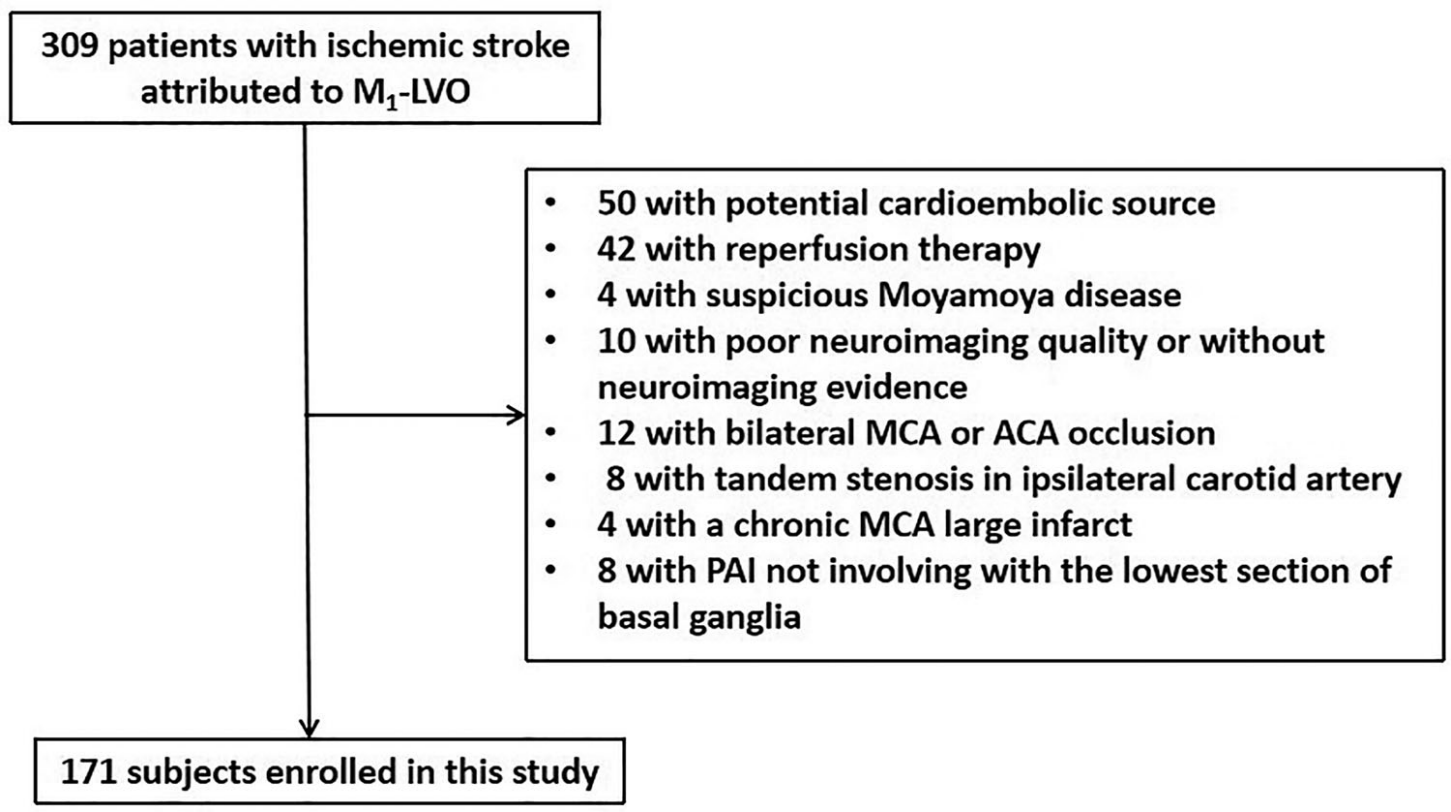
**Flow diagram of subjects’ enrollment**. ACA, anterior cerebral artery; LVO, large vessel occlusion; MCA, middle cerebral artery; PAI, perforating artery infarction.

A total of 309 patients with acute or subacute ischemic stroke due to M1‐LVO were screened. Among these patients, 50 with potential cardioembolic source, 42 with intravenous thrombolysis and (or) endovascular treatment, 4 with suspicious Moyamoya disease, 10 with poor neuroimaging quality or without neuroimaging evidence, 12 with bilateral MCA or ACA occlusion, 8 with tandem stenosis in ipsilateral carotid artery, 4 with a chronic large infarct within the territory of MCA, and 8 with PAI not involving with the lowest section of basal ganglia, these patients were all excluded. Finally, 171 participants were enrolled in this study, 98 from Affiliated Hospital of Jiangsu University and 73 from Siyang Hospital (Figure 5).

### 3.1 Baseline Data

The median age of 171 participants was 71.0 years (62.0, 77.0 years), and 95 (55.6%) participants were male. Of all participants, 31 (19.9%) were with subclassification of AAE, 32 (20.5%) with large infarct, 93 (59.6%) with BZI, and 15 (8.8%) with PAI. The mean proximal diameter of cMCA‐M_1_ segment was of 2.58 ± 0.61 mm, the median distal diameter of 2.08 mm (1.74, 2.45 mm), and the median arc length and chord length were of 22.73 mm (18.91, 27.06 mm) and 19.66 mm (16.42, 23.46 mm), respectively. The tortuosity index of cMCA‐M_1_ segment had a median of 14.95% (7.97, 22.29%). Ninety‐one (53.2%) patients suffered from a poor clinical outcome at discharge (Table S1).

### 3.2 The Comparison of Clinical Outcomes Among Infarct Patterns

Initial NIHSS (*p* < 0.001), the arc length (*p* < 0.001), chord length (*p* = 0.005), and tortuosity index (*p* = 0.002) of cMCA and the rate of poor outcome (*p* < 0.001) were significantly different among the four patterns in univariable analyses (Table 1).

**TABLE 1:** The univariable analyses of clinical characteristics among different infarct patterns.

Clinical characteristics	AAE (*n* = 31)	Large infarct (*n* = 32)	PAI (*n* = 15)	BZI (*n* = 93)	*p* value
Male, *n* (%)	17 (54.8)	18 (56.3)	11 (73.3)	49 (52.7)	0.52
Age (year), median (IQR)	71.0 (63.0, 80.0)	74.5 (68.0, 79.0)	69.0 (62.0, 77.0)	69.0 (62.0, 77.0)	0.067
Hypertension, *n* (%)	22 (71.0)	25 (78.1)	13 (86.7)	68 (73.1)	0.67
Diabetes mellitus, *n* (%)	8 (25.8)	6 (18.8)	2 (13.3)	25 (26.9)	0.65
Smoking, *n* (%)	13 (41.9)	9 (28.1)	8 (53.3)	33 (35.5)	0.36
Previous stroke, *n* (%)	14 (45.2)	7 (21.9)	3 (20.0)	24 (25.8)	0.15
SBP (mmHg), mean ± SD	147.7 ± 22.1	149.6 ± 34.0	159.9 ± 23.9	150.4 ± 18.5	0.40
DBP (mmHg), mean ± SD	82.0 ± 12.0	81.8 ± 13.6	89.9 ± 10.8	83.6 ± 12.6	0.18
TC (mmol/L), mean ± SD	4.38 ± 0.99	4.94 ± 1.17	4.46 ± 1.04	4.82 ± 1.20	0.15
TG (mmol/L), median (IQR)	1.35 (0.85, 2.24)	1.27 (1.02, 1.59)	1.41 (0.96, 1.71)	1.34 (0.98, 1.87)	0.87
LDL‐C (mmol/L), mean ± SD	2.41 ± 0.69	2.90 ± 0.96	2.50 ± 0.94	2.72 ± 0.91	0.13
HbA1c (%), median (IQR)	6.30 (5.70, 7.64)	6.25 (5.50, 7.88)	5.80 (5.50, 6.80)	6.00 (5.55, 7.05)	0.53
Hcy (mmol/L), median (IQR)	15.20 (10.80, 19.49)	11.87 (8.34, 17.10)	14.13 (12.60, 17.50)	12.40 (8.30, 17.68)	0.24
Platelet count (×10^9^/L), mean ± SD	217.0 ± 68.7	195.8 ± 63.9	188.0 ± 47.7	212.4 ± 58.5	0.26
cMCA morphology					
Proximal diameter (mm), mean ± SD	2.64 ± 0.57	2.43 ± 0.45	2.82 ± 0.52	2.56 ± 0.66	0.19
Distal diameter (mm), median (IQR)	2.30 (1.66, 2.46)	2.07 (1.75, 2.51)	2.33 (2.03, 2.51)	2.00 (1.67, 2.42)	0.27
Proximal to distal diameter ratio, median (IQR)	1.28 (1.05, 1.43)	1.13 (0.96, 1.34)	1.22 (1.14, 1.34)	1.24 (1.09, 1.54)	0.20
Arc length (mm), median (IQR)	21.66 (16.68, 26.24)	18.59 (16.13, 23.18)	25.38 (21.41, 27.93)	25.25 (20.59, 29.56)	<0.001^*^
Chord length (mm), median (IQR)	19.01 (15.46, 22.17)	17.68 (13.62, 20.51)	19.78 (18.05, 25.32)	20.61 (17.03, 24.43)	0.005^*^
Tortuosity index (%), median (IQR)	9.26 (6.75, 18.81)	10.43 (5.49, 18.96)	12.87 (6.57, 22.09)	17.45 (10.28, 30.51)	0.002^*^
ICAD in cMCA, *n* (%)	5 (16.1)	4 (12.5)	1 (6.7)	18 (19.4)	0.67
Initial NIHSS (point), median (IQR)	2.0 (1.0, 4.0)	9.0 (5.0, 13.0)	3.0 (1.0, 4.0)	3.0 (2.0, 5.0)	<0.001^*^
Poor outcome, *n* (%)	6 (19.4)	29 (90.6)	4 (26.7)	52 (55.9)	<0.001^*^

Abbreviations: AAE, artery‐to‐artery embolism; BZI, borderzone infarct; cMCA, contralateral middle cerebral artery; DBP, diastolic blood pressure; HbA1c, glycosylated hemoglobin; Hcy, homocysteine; ICAD, intracranial atherosclerotic disease; LDL‐C, low‐density lipoprotein cholesterol; NIHSS, National Institutes of Health Stroke Scale; PAI, perforating artery infarction; SBP, systolic blood pressure; TC, total cholesterol; TG, triglyceride.

^*^ *p* < 0.05 was considered statistically significant.

After adjusting for age, sex, and common clinical factors with a *p* < 0.2 in univariable analyses, including a history of previous stroke, diastolic blood pressure at admission, levels of total cholesterol and low‐density lipoprotein cholesterol, initial NIHSS, and morphological parameters of cMCA‐M_1_ segment including proximal diameter and tortuosity index, the risks of poor outcome in BZI (odds ratio [OR] = 5.51, 95% confidence interval [CI] = 1.71–17.78, *p* = 0.004) and large infarct (OR = 10.92, 95% CI = 2.01–59.27, *p* = 0.006) groups were higher than in AAE group (Table 2).

**TABLE 2:** The association between the infarct patterns and poor outcome.

Infarct pattern	Poor outcome			
	Model 1^a^		Model 2^b^	
	Adjusted OR	*p* value	Adjusted OR	*p* value
AAE	Ref.	/	Ref.	/
PAI	1.26 (0.25–6.32)	0.78	1.42 (0.28–7.26)	0.68
BZI	4.83 (1.54–15.10)	0.007^*^	5.51 (1.71–17.78)	0.004^*^
Large infarct	11.33 (2.09–61.36)	0.005^*^	10.92 (2.01–59.27)	0.006^*^

Abbreviations: AAE, artery‐to‐artery embolism; BZI, borderzone infarct; OR, odds ratio; PAI, perforating artery infarction.

^a^Adjusting for common factors, including age, sex, previous stroke, diastolic blood pressure at admission, the levels of total cholesterol and low‐density lipoprotein cholesterol, and initial NIHSS.

^b^Adjusting for common factors plus proximal diameter and tortuosity index of cMCA trunk.

^*^ *p* < 0.05 was considered statistically significant.

### 3.3 The Associations Between BZI and the Morphological Parameters

In the univariable analyses, three parameters, including the arc length, the chord length, and the tortuosity index of cMCA‐M_1_ segment, were significantly different among the four groups. After adjusting for age, sex, abovementioned common clinical factors, and the proximal diameter, the incidences of elevated arc length (OR = 2.47, 95% CI = 1.02–5.97, *p* = 0.045) and tortuosity index (OR = 2.85, 95% CI = 1.13–7.18, *p* = 0.026) in BZI groups are significantly higher than in other three groups. The chord lengths among these four groups were comparable (Table 3).

**TABLE 3:** The associations between the infarct patterns and the morphological parameters of cMCA.

Infarct pattern	Elevated level of cMCA morphological parameters					
	Arc length		Chord length		Tortuosity index	
	Adjusted OR^a^	*p*	Adjusted OR^a^	*p*	Adjusted OR^a^	*p*
AAE	Ref.	/	Ref.	/	Ref.	/
Large infarct	0.66 (0.21–2.12)	0.49	0.66 (0.21–2.08)	0.48	1.30 (0.36–4.71)	0.69
PAI	2.45 (0.84–7.28)	0.099	1.57 (0.58–4.26)	0.38	2.03 (0.60–6.90)	0.26
BZI	2.47 (1.02–5.97)	0.045^*^	1.63 (0.71–3.74)	0.25	2.85 (1.13–7.18)	0.026^*^

Abbreviations: AAE, artery‐to‐artery embolism; BZI, borderzone infarct; cMCA, contralateral middle cerebral artery; OR, odds ratio; PAI, perforating artery infarction.

^a^Adjusting for age, sex, previous stroke, diastolic blood pressure at admission, total cholesterol, low‐density lipoprotein cholesterol, initial NIHSS, and proximal diameter of cMCA.

^*^ *p* < 0.05 was considered statistically significant.

### 3.4 Correlation Between Large Infarct and Morphological Parameters Among Subjects With a Lower Tortuosity Index

Patients with BZI were excluded. The remaining 78 participants were dichotomized into non‐large‐infarct (*n* = 46) and large infarct (*n* = 32) groups. The levels of total cholesterol (*p* = 0.033) and low‐density lipoprotein cholesterol (*p* = 0.021), the proximal diameter (*p* = 0.027), arc length (*p* = 0.009), and chord length (*p* = 0.025) of cMCA‐M_1_ segment were significantly different, and the tortuosity index was comparable between the two groups (Table S2).

After adjusting for age, sex, and factors with a *p* < 0.2 in univariable analyses including levels of total cholesterol, low‐density lipoprotein cholesterol, and homocysteine, the proximal diameter (OR = 0.22, 95% CI = 0.07–0.72, *p* = 0.012), arc length (OR = 0.88, 95% CI = 0.78–0.98, *p* = 0.018), and chord length (OR = 0.87, 95% CI = 0.77–0.995, *p* = 0.042) of cMCA‐M_1_ segment were all negatively associated with the onset of large infarct (Table 4).

**TABLE 4:** The associations between the morphological parameters of cMCA and the onset of large infarct.

cMCA morphology	Large infarct			
	Crude OR	*p* value	Adjusted OR^a^	*p* value
Proximal diameter	0.35 (0.13–0.91)	0.032^*^	0.22 (0.07–0.72)	0.012^*^
Arc length	0.89 (0.82–0.98)	0.016^*^	0.88 (0.78–0.98)	0.018^*^
Chord length	0.89 (0.79–0.99)	0.030^*^	0.87 (0.77–0.995)	0.042^*^

Abbreviation: cMCA, contralateral middle cerebral artery.

^a^Adjusting for age, sex, total cholesterol, low‐density lipoprotein cholesterol, and homocysteine.

^*^ *p* < 0.05 was considered statistically significant.

The cMCA‐M_1_ morphological parameters between AAE and PAI groups were with no differences (Table S3).

## 4 Discussion

In this study, we found that in patients with ischemic stroke caused by M_1_‐ICAD‐LVO, the risk of poor outcomes increased by approximate 4.5‐fold and 10‐fold in BZI and large infarct compared to AAE, respectively; the arc length and tortuosity index of cMCA‐M_1_ segment in BZI were the highest among the four infarct patterns; the proximal diameter, arc, and chord length of cMCA‐M_1_ segment were negatively associated with the onset of large infarct.

Among the four infarct patterns attributed to the M_1_‐ICAD‐LVO, the rate of poor outcome of large infarct was the highest and up to 90%, which was in accordance with previous works (Yoshimura et al. 2022; Sarraj et al. 2023; Huo et al. 2023). However, the rate of poor outcome of BZI was 55.9%, far higher than previously reported (Yong et al. 2006). The disparity of clinical outcomes between the two studies maybe due to that the stenotic degree of culprit vessel in the present study (all occlusion) is greater than in the previous work (from 50% to occlusion), whereas the severer stenosis of culprit vessel determines a poorer clinical outcome (Lau et al. 2013). After adjusting for confounders in the multivariable analyses, the incidence of poor outcome was the highest in large infarct, followed by BZI, and was lowest both in PAI and AAE in this study, suggesting that the risk of poststroke disability of large infarct and BZI patterns was higher than AAE and PAI in the context of MCA trunk occlusion. The extensive loss of eloquent brain matter and substantial poststroke neuroinflammation are the probable reasons of the poor outcome of patients with large infarct (Bai et al. 2024); whereas for patients with BZI, the major pathogenesis is hypoperfusion, which could thereafter decrease the blood supply for oligemic tissue surrounding the ischemic penumbra and introduce with the subsequent infarct (Alawneh et al. 2009). These are the potential pathophysiological reasons for the poor outcomes of patients with large infarct or BZI. The relative favorable clinical outcomes of AAE and PAI may be related to the limited volumes of the ischemic core and penumbra.

Owing to the heterogeneous clinical outcomes of various infarct patterns in the context of M_1_‐ICAD‐LVO, we explored the vascular morphological features associated with these infarct patterns in order to roughly understand the pathogenesis underlying these patterns. We found in the univariable analyses that the arc length and tortuosity index of BZI were the largest among the four patterns, which was validated in the further multivariable analyses after adjusting for confounders. This indicates that a long and tortuous MCA trunk seems to correlate with the onset of BZI. However, the underlying mechanisms by which this vascular morphological characteristic is associated with BZI remain unclear. We speculate that the underlying mechanisms may be as follows: First, as the upstream vessel of the cerebral small vessels, the major intracranial artery with a large tortuosity could not only impair the intraluminal hemodynamics (Ha et al. 2023) but also cause the formation of cerebral small vessel disease (Brisset et al. 2013) and simultaneously affect the blood flow within these small vessels (Shang et al. 2022); second, the presence of small vessel disease could thereafter result in insufficient collaterals recruitments (Lin et al. 2020). The abovementioned pathophysiologies are detrimental to the blood supply for the territory of occlusive MCA, which is the probable mechanism underlying the onset of BZI in patients with M_1_‐ICAD‐LVO.

The distinctive cMCA‐M_1_ segment morphological parameters of patients with BZI were firstly identified; thus, these patients were excluded from the further analyses. Because of the poorest clinical outcomes of large infarct among the four patterns, so we attempted to find out the cMCA‐M_1_ segment morphological features of this category of patients. The morphological parameters of cMCA‐M_1_ segment were comparable among patients with AAE, PAI, and large infarct; we thereafter recategorized these patients into two groups: large infarct and non‐large‐infarct groups. Interestingly, subjects of the two groups had significantly different proximal diameters, arc lengths, and chord lengths of cMCA‐M_1_ segment which were all corroborated to be negatively associated with the onset of large infarct in multivariable regression analyses. This indicates that an ICAD‐MCA trunk with a small proximal lumen and a short length is possibly susceptible to the onset of large infarct. A previous work reported that a short vessel was correlated with a large infarct volume (Li et al. 2024), which supported the result of this study, but the mechanism was still unclear. We speculate that an MCA trunk with a thin lumen and a short length is prone to be instantly blocked by rapid growing and large clots, leading to the formation of large infarct before effective compensation from the collaterals.

The vascular morphological parameters of cMCA‐M_1_ segment were identical between AAE and PAI patterns, with a large proximal diameter and a low tortuosity. An MCA trunk with a low tortuosity maybe associated with good ipsilateral collaterals recruitments, and the incidence of large infarct in case of acute occlusion in this trunk is relatively low. However, a large luminal diameter is reported to be correlated with high plaque structural stress (Teng et al. 2014) which would cause large necrotic core (Huang et al. 2001) upregulated expression of matrix metalloproteinase and macrophage aggregation (Lee et al. 1996; Hallow et al. 2009) in the atherosclerotic plaque. Therefore, the elevated plaque structural stress is likely to be a pivotal factor mediating the plaque rupture, thrombosis, and the ultimate vascular events (Richardson et al. 1989). If the orifice of a perforating artery is completely blocked in the process of plaque rupture or thrombosis, the infarct pattern of PAI is probably to occur because that the perforating artery is an end‐artery and lack of collaterals (Rocha and Jovin 2017). The onset of AAE maybe attributed to the blockage of the end of cortical small vessel or perforating artery by the debris from vulnerable plaque or embolus.

This study has certain clinical significance for M_1_‐ICAD patients who have a high risk of acute occlusion and subsequent ischemic stroke and could provide preliminary risk stratification for these patients and guide the clinical administrated strategies of subgroups with different potential future infarct patterns. First, M_1_‐ICAD patients with a long and a tortuous cMCA‐M_1_ segment are susceptible to the onset of BZI and they may not benefit from intensive control of blood pressure (Yamauchi et al. 2013). Second, for M_1_‐ICAD patients with a relatively straight, thin, and short cMCA‐M_1_ segment, the risk of large infarct is relatively high. A recent study has reported that the balloon angioplasty combined with medical management is more superior than sole medical management for patients with intracranial symptomatic ICAD (Sun et al. 2024). Whether endovascular treatment, such as balloon angioplasty, could improve the prognoses of asymptomatic M_1_‐ICAD patients who have a high risk of large infarct is needed to be validated in subsequent clinical trails. Lastly, for those with a straight, thick, and moderate long cMCA‐M_1_ segment, the culprit vessel is susceptible to the formation of vulnerable plaque, which probably leads to the onset of AAE or PAI; thus, the intensive statin therapy seems to be important (Jing et al. 2018).

This study has some limitations. First, despite as a double‐center study, this study has a relatively small sample size and the nature of retrospective study. Whether there is a causal relationship between the cMCA‐M_1_ morphological features and the onset of different infarct patterns caused by M_1_‐ICAD‐LVO needs to be demonstrated in further prospective studies. Second, the morphological parameters of the occlusive culprit vessel could not be measured, which was surrogated by the parameters of cMCA. We also excluded patients who achieved recanalization of the occluded vessel following mechanical thrombectomy due to concerns that the thrombectomy procedure might influence the stroke patterns. However, previous research has shown no significant differences in the morphological features between the bilateral MCAs in the same individual (Kim et al. 2015). Therefore, based on the morphological features of the cMCA, we preliminarily explored the pathogenesis of various stroke patterns. We plan to formulate subsequent animal experiments to verify our hypotheses. Third, the infarct volume is likely to be associated with the clinical outcomes of ischemic stroke. However, we did not calculate the infarct volume of each participant because the infarct lesions were scattered and the volume was difficult to be calculated in some stroke patterns such as BZI and AAE. In further study, we would employ some special software to measure the infarct volume of stroke patients, in order to validate the results of this study. Finally, we utilized magnetic resonance or CT angiography to assess the vascular morphology of the subjects. This may influence the study results. However, implementing a uniform imaging protocol in clinical practice is often unfeasible. For example, some patients had metallic implants precluding magnetic resonance angiography, whereas others with renal impairment were unsuitable for CT angiography. Thus, the utilization of diverse imaging techniques for cerebrovascular evaluation is an unavoidable limitation in real‐world settings.

## 5 Conclusion

Among the infarct patterns caused by M_1_‐ICAD‐LVO, the clinical outcome was poorest in large infarct, followed by BZI, and the outcomes of AAE and PAI were relatively favorable. The elevated tortuosity and arc length of cMCA‐M_1_ segment were associated with the onset of BZI, whereas small proximal diameter and short arc length were suggestive of the possible onset of large infarct in patients with cMCA of low tortuosity. This study contributed to provide some potential imaging indicators for the future infarct patterns of M_1_‐ICAD patients with a high risk of stroke and also provide guidance for their clinical managements.

## Author Contributions

**ZhiRong Cai**: conceptualization, writing – original draft, methodology. **Yuan Chen**: writing – original draft, investigation, data curation. **ShaoQing Pei**: data curation, investigation, software. **Yue He, YaNan Zhu, Rui Zhang, and JingWei Lin**: data curation, investigation. **Yi Yang**: funding acquisition, resources, supervision, and validation. **Ying Zhu**: supervision and validation.

## Supporting information




**Supplementary Material**: brb371226‐sup‐0001‐SuppMat.docx

## Data Availability

All data generated or analyzed during this study could be acquired from the corresponding author through e‐mail.
