# Supplementary material for: The Correlations of Clinical Outcomes and Vascular Morphology With Infarct Patterns in Middle Cerebral Arterial Occlusion
Source: Brain Behav. 2026 Jan 28;16(2):e71226. doi: 10.1002/brb3.71226 (PMC12848514; doi:10.1002/brb3.71226)
Supplement: Supplementary file 1 — Supplementary Material: brb371226‐sup‐0001‐SuppMat.docx [file BRB3-16-e71226-s001.docx]

Supplementary Table S1 The clinical characteristics of all subjects.

| Clinical characteristics | All subjects  (n=171) |
| --- | --- |
| Male, n (%) | 95 (55.6) |
| Age (year), median (IQR) | 71.0 (62.0, 77.0) |
| Hypertension, n (%) | 128 (74.9) |
| Diabetes mellitus, n (%) | 41 (24.0) |
| Smoking, n (%) | 63 (36.8) |
| Previous stroke, n (%) | 48 (28.1) |
| SBP (mmHg), mean ± SD | 150.6 ± 23.2 |
| DBP (mmHg), mean ± SD | 83.5 ± 12.6 |
| TC (mmol/L), mean ± SD | 4.73 ± 1.16 |
| TG (mmol/L), median (IQR) | 1.34 (0.97, 1.84) |
| LDL-C (mmol/L), mean ± SD | 2.68 ± 0.89 |
| HbA1c (%), median (IQR) | 6.05 (5.60, 7.20) |
| Homocysteine (mmol/L), median (IQR) | 12.97 (8.90, 17.77) |
| Platelet count (×10^9^/L), mean ± SD | 208.0 ± 60.9 |
| Infarct pattern |  |
| AAE, n (%) | 31 (19.9) |
| Large infarct, n (%) | 32 (20.5) |
| BZI, n (%) | 93 (59.6) |
| PAI, n (%) | 15 (8.8) |
| Initial NIHSS (point), median (IQR) | 4.0 (2.0, 6.0) |
| cMCA morphology |  |
| Proximal diameter (mm), mean ± SD | 2.58 ± 0.61 |
| Distal diameter (mm), median (IQR) | 2.08 (1.74, 2.45) |
| Proximal to Distal diameter ratio, median (IQR) | 1.23 (1.07, 1.50) |
| Arc length (mm), median (IQR) | 22.73 (18.91, 27.06) |
| Chord length (mm), median (IQR) | 19.66 (16.42, 23.46) |
| Tortuosity index (%), median (IQR) | 14.95 (7.97, 22.29) |
| ICAD in cMCA, n (%) | 28 (16.4) |
| Poor outcome at discharge, n (%) | 91 (53.2) |

Abbreviations: SBP indicates systolic blood pressure; DBP, diastolic blood pressure; TC, total cholesterol; TG, triglyceride; LDL-C, low-density lipoprotein cholesterol; HbA1c, glycosylated hemoglobin; AAE indicates artery-artery embolism; BZI, borderzone infarct; PAI, perforating artery infarction; NIHSS, National Institutes of Health Stroke Scale; cMCA, contralateral middle cerebral artery; ICAD, intracranial atherosclerotic disease.

Supplementary Table S2 The univariable analyses of clinical characteristics between patients with large infarct and with non-large-infarct.

Abbreviations: SBP indicates systolic blood pressure; DBP, diastolic blood pressure; TC, total cholesterol; TG, triglyceride; LDL-C, low-density lipoprotein cholesterol; HbA1c, glycosylated hemoglobin; cMCA, contralateral middle cerebral artery; ICAD, intracranial atherosclerotic disease.

* *p* < 0.05 was considered statistically significant.

| Clinical characteristics | Non-large infarct  (n=46) | Large infarct  (n=32) | P-value |
| --- | --- | --- | --- |
| Male, n (%) | 28 (60.9) | 18 (56.3) | 0.68 |
| Age (year), median (IQR) | 71.0 (62.8, 79.3) | 74.5 (68.0, 79.0) | 0.41 |
| Hypertension, n (%) | 35 (76.1) | 23 (71.9) | 0.68 |
| Diabetes mellitus, n (%) | 12 (26.1) | 6 (18.8) | 0.45 |
| Smoking, n (%) | 16 (34.8) | 10 (31.3) | 0.75 |
| Previous stroke, n (%) | 10 (21.7) | 10 (31.3) | 0.34 |
| SBP (mmHg), mean ± SD | 151.7 ± 23.2 | 149.6 ± 34.0 | 0.75 |
| DBP (mmHg), mean ± SD | 84.5 ± 12.1 | 81.8 ± 13.6 | 0.35 |
| TC (mmol/L), mean ± SD | 4.40 ± 1.00 | 4.94 ± 1.17 | 0.033^*^ |
| TG (mmol/L), median (IQR) | 1.38 (9.32, 2.18) | 1.27 (1.02, 1.59) | 0.51 |
| LDL-C (mmol/L), mean ± SD | 2.44 ± 0.77 | 2.90 ± 0.96 | 0.021^*^ |
| HbA1c (%), median (IQR) | 6.15 (5.67, 7.15) | 6.25 (5.50, 7.88) | 0.93 |
| Hcy (mmol/L),median (IQR) | 14.81 (11.61, 19.42) | 11.87 (8.34, 17.09) | 0.057 |
| Platelet count (×10^9^/L), mean ± SD | 207.5 ± 63.6 | 195.8 ± 63.9 | 0.43 |
| cMCA morphology |  |  |  |
| Proximal diameter (mm), mean ± SD | 2.70 ± 0.56 | 2.43 ± 0.44 | 0.027^*^ |
| Distal diameter (mm), median (IQR) | 2.32 (1.75, 2.48) | 2.07 (1.75, 2.51) | 0.66 |
| Proximal to Distal diameter ratio,  median (IQR) | 1.24 (1.07, 1.42) | 1.13 (0.96, 1.34) | 0.082 |
| Arc length (mm), median (IQR) | 22.14 (19.00, 26.27) | 18.59 (16.13, 23.18) | 0.009^*^ |
| Chord length (mm), median (IQR) | 19.60 (16.30, 22.51) | 17.68 (13.62, 20.51) | 0.025^*^ |
| Tortuosity index (%), median (IQR) | 12.25 (6.70, 19.56) | 10.43 (5.49, 18.96) | 0.50 |
| ICAD in cMCA, n (%) | 6 (13.0) | 4 (12.5) | 0.99 |

Supplementary Table S3 The univariable analyses of clinical characteristics between patients with AAE and with PAI

Abbreviations: AAE indicates artery-artery embolism; PAI, perforating artery infarction; SBP, systolic blood pressure; DBP, diastolic blood pressure; TC, total cholesterol; TG, triglyceride; LDL-C, low-density lipoprotein cholesterol; HbA1c, glycosylated hemoglobin; Hcy, homocysteine; cMCA, contralateral middle cerebral artery; ICAD, intracranial atherosclerotic disease.

| Clinical characteristics | AAE (n=31) | PAI (n=15) | P-value |
| --- | --- | --- | --- |
| Male, n (%) | 17 (54.8) | 11 (73.3) | 0.23 |
| Age (year), median (IQR) | 71.0 (63.0, 80.0) | 69.0 (62.0, 77.0) | 0.72 |
| Hypertension, n (%) | 22 (71.0) | 13 (86.7) | 0.42 |
| Diabetes mellitus, n (%) | 8 (25.8) | 2 (13.3) | 0.56 |
| Smoking, n (%) | 13 (41.9) | 8 (53.3) | 0.47 |
| Previous stroke, n (%) | 14 (45.2) | 3 (20.0) | 0.097 |
| SBP (mmHg), mean ± SD | 147.7 ± 22.1 | 159.9 ± 23.9 | 0.093 |
| DBP (mmHg), mean ± SD | 82.0 ± 12.0 | 89.9 ± 10.8 | 0.036^*^ |
| TC (mmol/L), mean ± SD | 4.38 ± 0.99 | 4.46 ± 1.04 | 0.80 |
| TG (mmol/L), median (IQR) | 1.35 (0.85, 2.24) | 1.41 (0.96, 1.71) | 0.75 |
| LDL-C (mmol/L), mean ± SD | 2.41 ± 0.69 | 2.50 ± 0.94 | 0.73 |
| HbA1c (%), median (IQR) | 6.30 (5.70, 7.64) | 5.80 (5.50, 6.80) | 0.15 |
| Hcy (mmol/L),median (IQR) | 15.20 (10.80, 19.49) | 14.13 (12.60, 17.50) | 0.64 |
| Platelet count (×10^9^/L), mean ± SD | 217.0 ± 68.7 | 188.0 ± 47.7 | 0.15 |
| cMCA morphology |  |  |  |
| Proximal diameter (mm), mean ± SD | 2.64 ± 0.57 | 2.82 ± 0.52 | 0.29 |
| Distal diameter (mm), median (IQR) | 2.30 (1.66, 2.46) | 2.33 (2.03, 2.51) | 0.29 |
| Proximal to Distal diameter ratio,  median (IQR) | 1.28 (1.05, 1.43) | 1.22 (1.14, 1.34) | 0.86 |
| Arc length (mm), median (IQR) | 21.66 (16.68, 26.24) | 25.38 (21.41, 27.93) | 0.089 |
| Chord length (mm), median (IQR) | 19.01 (15.46, 22.17) | 19.78 (18.05, 25.32) | 0.19 |
| Tortuosity index (%), median (IQR) | 9.26 (6.75, 18.81) | 12.87 (6.57, 22.09) | 0.43 |
| ICAD in cMCA, n (%) | 5 (16.1) | 1 (6.7) | 0.65 |
